# The Synthesis of Materials with a Hierarchical Structure Based on Tin Dioxide

**DOI:** 10.3390/nano14221813

**Published:** 2024-11-13

**Authors:** Ekaterina Bondar, Elena Dmitriyeva, Igor Lebedev, Anastasiya Fedosimova, Aigul Shongalova, Sayora Ibraimova, Ainagul Kemelbekova, Ulzhalgas Issayeva, Bagdat Rakymetov, Bedelbek Nurbaev

**Affiliations:** Institute of Physics and Technology, Satbayev University, Ibragimov 11, Almaty 050013, Kazakhstan; lebedev692007@yandex.ru (I.L.); ananastasia@list.ru (A.F.); shongalova.aigul@gmail.com (A.S.); sayara_ibraimova@mail.ru (S.I.); a.kemelbekova@sci.kz (A.K.); ulyajan_1603@mail.ru (U.I.); bagdat_r@mail.ru (B.R.); bedel.armia.99@gmail.com (B.N.)

**Keywords:** nanostructures, thin films, sol–gel chemistry, X-ray diffraction, crystal structure

## Abstract

This article presents the results of the formation of hierarchical micro–nano structures in nanostructured tin dioxide films obtained from the lyophilic film-forming system SnCl_4_/EtOH/NH_4_OH. The classification of the shape and size of the synthesized structures, in relation to the pH of the solution, is presented. Measurements were carried out on an X-ray diffractometer to study the crystal structure of the samples analyzed. It was found that SnO_2_ and NH_4_Cl crystallites participate in the formation of the synthesized hierarchical structures. It is shown that the mechanism of the formation of hierarchical structures depends on the amount of ammonium hydroxide added. This makes it possible to control the shape and size of the synthesized structures by changing the ratio of precursors.

## 1. Introduction

Metal oxides (SnO_2_, ZnO, TiO_2_, In_2_O_3_, etc.) represent a rather interesting class of materials for fundamental research, since they have a wide range of electrical and optical properties [1].

Composite systems based on tin dioxide, as a functional material, have many applications [2,3,4]. The high chemical homogeneity of the obtained products allows the use of SnO_2_ coatings as three-dimensional macroporous anodes in lithium-ion batteries [5], as active layers in gas analysis equipment, as protective coatings against corrosion, etc. [6,7,8].

It should be noted that thin films of undoped SnO_2_ quickly see their properties deteriorate when heated above 500 °C since they are in a metastable phase. Doping SnO_2_ with various chemical elements (F, Sb, Ga, Pd, Fe, Si, Ni, Gr, Au, etc.) can significantly change its electrical, optical, and gas-sensitive properties, as well as any other properties [9,10,11,12,13]. It also expands the application possibilities of thin films made of tin dioxide. In addition, the properties of these films can be improved by treating them with various types of plasma [14,15].

One of the distinctive characteristics of nanosized tin dioxide films is their combination of high electrical conductivity with transparency in the visible and ultraviolet radiation regions. Oxygen non-stoichiometry has a direct effect on the functionality of oxides. In turn, the technology of oxide production and its subsequent processing have large roles in oxygen non-stoichiometry [16,17]. The pH of film-forming systems plays an important role in determining the structure and properties of the resulting films. Systems with a pH range of 7–11 are often considered in studies [18]. In this work, the pH range of 1.4–1.53 was studied. In this range, changes in light absorption and surface resistance are observed, so that there is a transition from the formation of surface SnO_2_ to the formation of bulk SnO_2_ [19]. The advantage of a comprehensive study is the dependence of the transparency of the samples and their resistance on the change in the pH of the film-forming system, which is very important for solar cells and gas sensors.

In recent years, a type of material that can be called functional hierarchical material has clearly emerged in the field of functional materials. Such materials consist of elements of several scales, which are organized in such a way that elements of a smaller scale are inserted into elements of a larger scale. The main mechanism for the formation of hierarchical nanomaterials is self-assembly. Materials with a hierarchical structure have significant potential for practical applications [20,21,22,23,24,25].

Hierarchical structures based on SnO_2_ are intensively studied, since they have a large surface area, high surface permeability, low density, low cost, environmental friendliness, and stable physical and chemical characteristics [26]. 

This work presents the possibility of synthesizing thermally stable hierarchical micro–nano structures with adjustable sizes, which depend on the ratio of ammonium ions to tin ions in the film-forming system SnCl_4_/EtOH/NH_4_OH, using sol–gel technology.

## 2. Materials and Methods

The following reagents were used to conduct an experiment on the synthesis of film-forming systems:

(1) Tin chloride pentahydrate powder SnCl_4_·5H_2_O;
(2) Ethyl alcohol C_2_H_5_OH (GOST 5962-13);
(3) Concentrated aqueous solution of ammonia NH_4_OH.

A total of 4 film-forming solutions were prepared using the sol–gel method. For the preparation of these solutions, the following components were used:

(1) m(SnCl_4.5_H_2_O) = 3.9072 g.
(2) V(C_2_H_5_OH) = 100 mL.
(3) V(NH_4_OH) = 0 mL; 0.4 mL; 0.8 mL; 1.6 mL.

SnCl_4_·5H_2_O powder was poured into a flask V = 100 mL and dissolved in ethyl alcohol V = 50 mL. Next, ethyl alcohol V = 25 mL was poured into a separate flask and the required amount of concentrated aqueous ammonia solution was added. The resulting solution was introduced into the original flask drop by drop. Then, the remaining volume of ethyl alcohol was poured in and mixed thoroughly, and the solution was sent to a dark place for a day to “mature”.

Table 1 shows the amount of added ammonia, the pH of the solution after maturation, and the ratio of ammonium ions to tin ions.

The following chemical reactions occur in the systems:

SnCl_4_ + 4C_2_H_5_OH → Sn(OH)_4_ + 4C_2_H_5_Cl (1)

SnCl_4_ + 4H_2_O → Sn(OH)_4_ + 4HCl (2)

T, °C Sn(OH)_4_ → SnO_2_ + 2H_2_O (3)

When interacting with a concentrated aqueous solution of ammonia, the following reactions occur:

SnCl_4_ + NH_4_OH → Sn(OH)_4_ + 4NH_4_Cl (4)

NH_4_OH + HCl → NH_4_Cl + H_2_O (5)

The synthesized samples were annealed at 400 °C for 15 min.

## 3. Results and Discussion

The synthesized samples were examined using an optical microscope, a Raman spectroscope Solver Spectrum (NT-MDT, Moscow, Russia), an X-ray diffractometer DRON-6 Burevestnik (St. Petersburg, Russia), and a scanning electron microscope (SEM) JEOL (JSM-6490LA, Tokyo, Japan). 

### 3.1. Optical Microscopy

**Figure 1:**
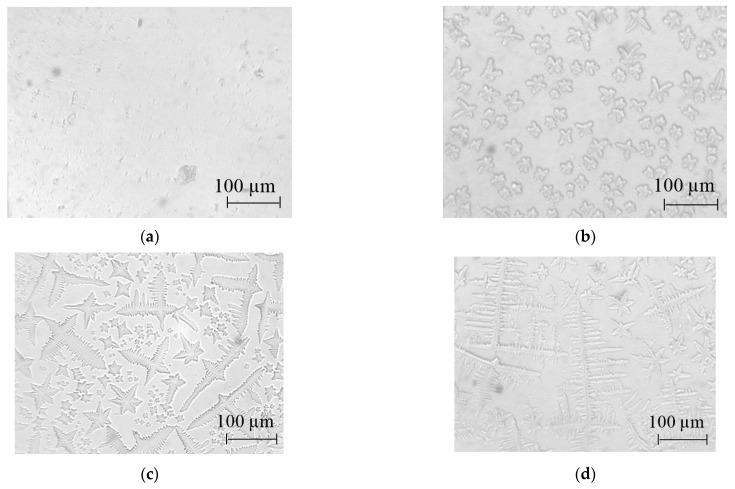
The structure of the film obtained from the film-forming system SnCl_4_/EtOH/NH_4_OH via the sol–gel method: (**a**) without adding NH_4_OH; (**b**) 0.4 mL NH_4_OH per 100 mL of solution; (**c**) 0.8 mL NH_4_OH per 100 mL of solution; (**d**) 1.6 mL NH_4_OH per 100 mL of solution.

To find non-transparent objects in the synthesized samples, studies were carried out using an MPE-11 optical microscope. Figure 1 shows photographs of the films obtained from the film-forming system SnCl_4_/EtOH/NH_4_OH using different ratios of ammonium ions to tin ions.

It can be seen in Figure 1 that, in the absence of ammonium hydroxide (Figure 1a), no regular structures are detected in the film. When ammonium ions are added, certain structures begin to form, the shape and size of which depend on the relative amount of tin ions N(Sn) and ammonium ions N(NH_4_).

At N(Sn) > N(NH_4_), structures resembling a six-petal flower are formed (Figure 1b). The average size of the synthesized structures reaches 40 µm.

At N(Sn) < N(NH_4_), the formation of cross-shaped structures predominates (Figure 1d). Their size is significantly higher than that of the flower-like structures and reaches more than 300 µm. 

When the number of tin ions is approximately the same as the number of ammonium ions, some mixed structures are obtained that resemble neither a cross-shaped structure nor a six-petal flower (Figure 1c).

### 3.2. Raman Spectroscopy

Raman spectra of the samples were obtained at room temperature, with a laser wavelength of 473 nm and a spectral resolution of 4 cm^−1^, using Solver Spectrum (NT-MDT, Moscow, Russia).

**Figure 2:**
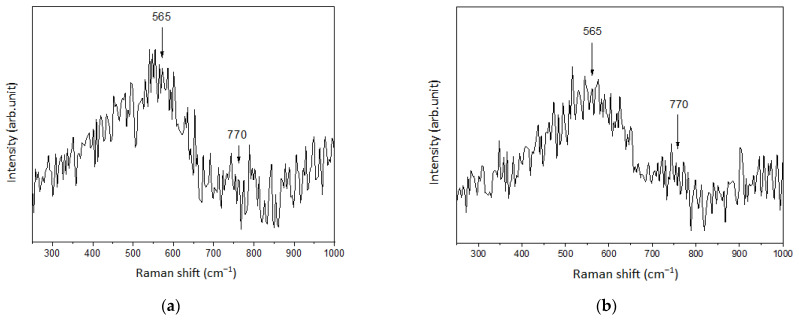
Raman spectra of the sample: (**a**) without adding NH_4_OH; (**b**) 1.6 mL NH_4_OH per 100 mL of solution.

Figure 2 shows the Raman spectra of the synthesized samples with and without the addition of NH_4_OH. The spectra of the samples show an intense broad band at 565 cm^−1^ and a less intense band at 770 cm^−1^. These bands can be ascribed to the vibrational modes of amorphous SnO_2_ according to [27].

The spectra are very similar and therefore do not allow us to identify any distinctive features in the formation of the synthesized films.

### 3.3. X-Ray Diffraction

**Figure 3:**
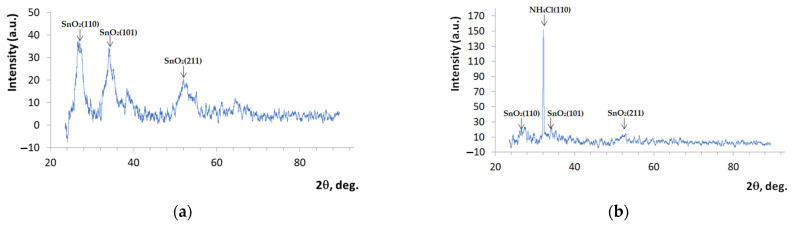
X-ray diffraction pattern of the crystal structure of the film obtained from the film-forming system SnCl_4_/EtOH/NH_4_OH by the sol–gel method, measured on a DRON-6 X-ray diffractometer: (**a**) sample without NH_4_OH; (**b**) sample with NH_4_OH.

To study the crystal structure of the films obtained, measurements were carried out on a DRON-6 X-ray diffractometer (Burevestnik, St. Petersburg, Russia). The data obtained were processed using the ASAS method [28]. The results are presented in Figure 3.

In Figure 3a, which shows the X-ray diffraction pattern of the sample without NH_4_OH addition, three clear peaks emerge from the crystallographic planes of tin dioxide SnO_2_(110), SnO_2_(101), and SnO_2_(211).

In Figure 3b, a peak emerging from the crystallographic plane of ammonium chloride NH_4_Cl(110) is visible. The signals from three crystallographic planes, namely, SnO_2_(110), SnO_2_(101), and SnO_2_(211), are also highlighted. Unfortunately, the SnO₂ peaks in the X-ray diffraction spectra shown in Figure 3b are not as clear as those shown in Figure 3a. This is explained by the texturing effect: the peaks from tin dioxide, which have a relatively low intensity, are superimposed on the peak from ammonium chloride, which is several times more intense. 

The average crystallite sizes were calculated using the Scherrer formula, which relates the crystallite sizes to the width of the diffraction peaks [29]. The SnO_2_ crystallite size is 3.6 nm, and the NH_4_Cl crystallite size is 109 nm.

**Figure 4:**
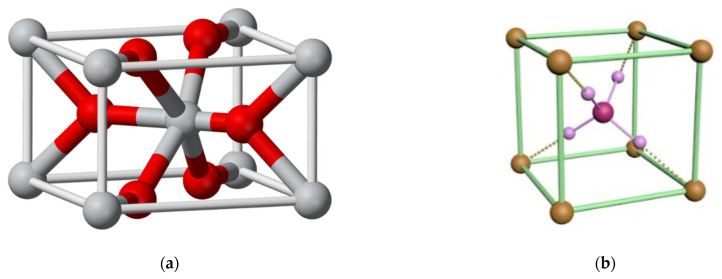
Elementary cells of (**a**) SnO_2_ and (**b**) NH_4_Cl.

Thus, SnO_2_ and NH_4_Cl crystallites participate in the formation of the synthesized hierarchical structures. In order to try to explain the correlation between the characterized samples and optical microscope images in relation to the amount of ammonium ions, we propose the consideration of the elementary cells of SnO_2_ and NH_4_Cl, which are presented in Figure 4.

The unit cell of SnO_2_, in which six tin atoms are bonded to nine oxygen atoms, visually resembles a six-petal flower. And the unit cell of NH_4_Cl resembles a cross of four hydrogen atoms attached to a nitrogen atom. It can be assumed that, at low NH_4_OH concentrations, the dominant basis for the formation of the synthesized hierarchical structures is the unit cells made of SnO_2_. At high NH_4_OH concentrations, a significant contribution is made by the unit cells of NH_4_Cl. 

**Figure 5:**
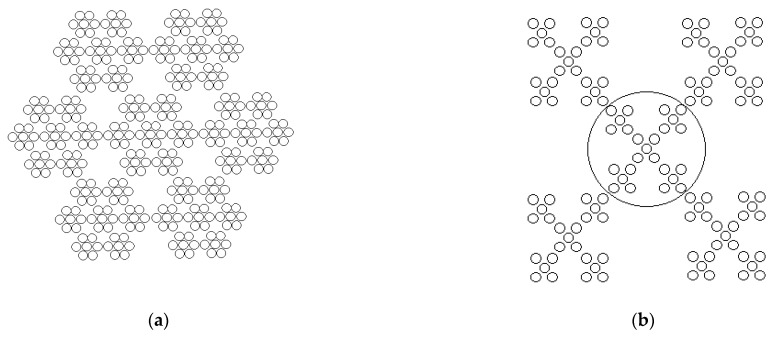
(**a**) Julien fractal aggregate; (**b**) two-dimensional disordered aggregate.

As an explanation for the association of the “flower” and “cross” morphologies on a scale of several micrometers in a single-crystal structure, the presence of a fractal construction of the structures shown in Figure 1b–d is assumed. As an example, Figure 5a shows a Julien fractal aggregate, in which a six-petal structural element forms a six-petal object of a larger size [30]. There is a certain initial particle in the center of the aggregate, and the other six particles are attached to it. In this case, all particles are identical, moving along the positive and negative directions of the three basis vectors of the lattice. The first stage of such an aggregate is an initial ensemble of seven particles. At the second stage, the same “flowers” are attached to six sides, and so on. That is, the main property of fractal aggregates is self-similarity [31,32,33]. Figure 5b shows a two-dimensional disordered aggregate, in which the cross morphology is formed from a cruciform structural element.

The addition of NH_4_OH significantly affects the structure of the films. Tin tetrachloride SnCl_4_ dissolves in ethyl alcohol C_2_H_5_OH. As a result, a highly dispersed colloidal system (sol) is formed. In colloidal systems, dispersed particles do not precipitate. In a suspended state, they are maintained by Brownian motion [34,35,36]. But unlike what occurs under the usual Brownian motion of particles, dispersed particles in colloidal solutions cannot meet under normal conditions, which is due to them having the same charge as the particles of the dispersed phase. The addition of ammonium hydroxide leads to the deformation of the electric layer of the dispersed phase. This accelerates the coagulation process. During sol coagulation, tin hydroxide Sn(OH)_4_ is formed, which has a gel-like structure. Unstable Sn(OH)_4_ decomposes with the formation of water and tin dioxide. In addition, as shown in Figure 3b, NH_4_Cl, which is formed during chemical reactions (4) and (5), is incorporated into the film’s structure. This leads to a change in the shape of the synthesized structures.

Thus, the mechanism of the formation of hierarchical structures depends on the amount of ammonium hydroxide added. This makes it possible to control the shape and size of the synthesized structures by changing the ratio of precursors.

### 3.4. Scanning Electron Microscopy (SEM)

**Figure 6:**
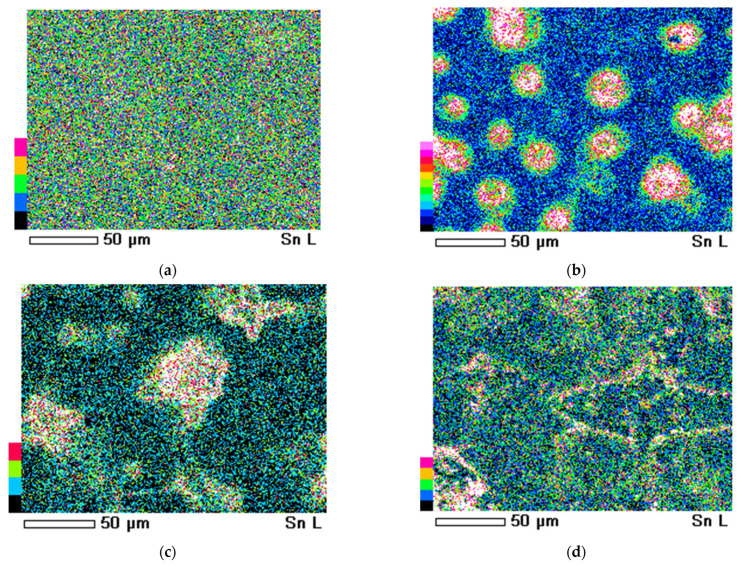
The mapping of samples obtained from the film-forming system SnCl_4_/EtOH/NH_4_OH with different additive contents: (**a**) without additive; (**b**) 0.4 mL NH_4_OH per 100 mL of solution; (**c**) 0.8 mL NH_4_OH per 100 mL of solution; (**d**) 1.6 mL NH_4_OH per 100 mL of solution.

To better understand the distribution of tin in the obtained samples, we mapped them using SEM. The results are shown in Figure 6. 

The gradation of the mapping scale is shown on the left in Figure 6, moving from black to red, pink, and crimson colors. This shows the level of tin content in the sample areas. As can be seen from Figure 6a, the film synthesized from the film-forming solution without additives shows a uniform distribution of tin throughout the sample, since the entire area of the analyzed sample is covered uniformly with multi-colored dots associated with the tin content scale. When forming structures resembling a six-petal flower (Figure 6b), the greatest accumulation of tin is observed precisely in the area of these structures. The middle of these structures is highlighted in crimson and red, with yellow and green colors seen along the edges. This indicates that, in these places, the concentration of tin is the highest. Conversely, the rest of the sample area is marked in black, blue, and light blue colors. This indicates the lower tin content in this area. With a further increase in the ammonia content in the solution (Figure 6c,d), a decrease in the tin content is observed near the cruciform structures. This confirms our assumption, outlined in Section 3.3, about the formation of hierarchical structures from SnO_2_ and NH_4_Cl crystallites. That is to say, the size and shape of the hierarchical structures depend on the relative amounts of tin ions and ammonium ions.

## 4. Conclusions

Thermally stable hierarchical micro–nano structures were synthesized from the film-forming system SnCl_4_/EtOH/NH_4_OH using sol–gel technology. The use of the film-forming system SnCl_4_/EtOH/NH_4_OH allows us to create hierarchical micro–nano structures with an adjustable (depending on the pH of the solution) size. The discovered relationship between the technological factors and the film’s structure has significant practical value for the formation of gas-sensitive layers of the material. To develop a better understanding of the tin distribution in the samples obtained, they were mapped. When forming structures resembling a six-petal flower, the greatest accumulation of tin is observed precisely in the area of these structures. With a further increase in the ammonia content in the solution, a decrease in the tin content is observed near the cruciform structures, which confirms our assumption about the formation of the synthesized hierarchical structures in such films being due to the presence of NH_4_Cl ions. The synthesized hierarchical structures may increase the gas sensitivity of the sensors of various gasses. These studies require further careful experiments. 

## Figures and Tables

**Table 1:** Ratio of ammonium ions to tin ions in film-forming systems.

V(NH_4_OH), mL	pH of Film-Forming Systems	Tin Ions in 100 mL (in Moles)	Ammonium Ions in 100 mL (in Moles)	Ammonium/Tin Ratio
0	1.40	0.011	0	0
0.4	1.44	0.011	0.005	0.455
0.8	1.46	0.011	0.01	0.909
1.6	1.49	0.011	0.02	1.818

## Data Availability

The original contributions presented in the study are included in the article, further inquiries can be directed to the corresponding author.
